# Circulating fatty acid profiles are associated with protein energy wasting in maintenance hemodialysis patients: a cross-sectional study

**DOI:** 10.1038/s41598-020-80812-1

**Published:** 2021-01-14

**Authors:** Ban-Hock Khor, Sharmela Sahathevan, Ayesha Sualeheen, Mohammad Syafiq Md Ali, Sreelakshmi Sankara Narayanan, Karuthan Chinna, Abdul Halim Abdul Gafor, Bak-Leong Goh, Ghazali Ahmad, Zaki Morad, Zulfitri Azuan Mat Daud, Pramod Khosla, Kalyana Sundram, Tilakavati Karupaiah, PaTCH Investigators, Boon Cheak Bee, Soo Kun Lim, Ravindran Visvanathan, Rosnawati Yahya, Sunita Bavanandan

**Affiliations:** 1 Department of Medicine, Faculty of Medicine, Universiti Kebangsaan Malaysia Medical Center, 56000 Kuala Lumpur, Malaysia; 2 Dietetics Program, Faculty of Health Sciences, Universiti Kebangsaan Malaysia, 50300 Kuala Lumpur, Malaysia; 3 Department of Nutrition and Dietetics, Faculty of Medicine and Health Sciences, Universiti Putra Malaysia, 43400 Seri Kembangan, Selangor Malaysia; 4 School of BioSciences, Faculty of Health and Medical Sciences, Taylor’s University, 47500 Subang Jaya, Selangor Malaysia; 5 School of Medicine, Faculty of Health and Medical Sciences, Taylor’s University, 47500 Subang Jaya, Selangor Malaysia; 6 Clinical Research Center, Hospital Serdang, 43000 Kajang, Selangor Malaysia; 7 Department of Nephrology, Hospital Kuala Lumpur, 53000 Kuala Lumpur, Malaysia; 8 National Kidney Foundation of Malaysia, 46100 Petaling Jaya, Selangor Malaysia; 9 Department of Nutrition and Food Sciences, Wayne State University, Detroit, MI 48202 USA; 10 Malaysia Palm Oil Council, 47301 Kelana Jaya, Malaysia; 11 Department of Nephrology, Hospital Selayang, 68100 Batu Caves, Selangor Malaysia; 12 Department of Nephrology, Universiti Malaya Medical Center, 59100 Kuala Lumpur, Malaysia

**Keywords:** Nutrition, Haemodialysis

## Abstract

The metabolic impact of circulating fatty acids (FAs) in patients requiring hemodialysis (HD) is unknown. We investigated the associations between plasma triglyceride (TG) FAs and markers of inflammation, insulin resistance, nutritional status and body composition. Plasma TG-FAs were measured using gas chromatography in 341 patients on HD (age = 55.2 ± 14.0 years and 54.3% males). Cross-sectional associations of TG-FAs with 13 markers were examined using multivariate linear regression adjusted for potential confounders. Higher levels of TG saturated fatty acids were associated with greater body mass index (BMI, *r* = 0.230), waist circumference (*r* = 0.203), triceps skinfold (*r* = 0.197), fat tissue index (*r* = 0.150), serum insulin (*r* = 0.280), and homeostatic model assessment of insulin resistance (*r* = 0.276), but lower malnutrition inflammation score (MIS, *r* =  − 0.160). Greater TG monounsaturated fatty acid levels were associated with lower lean tissue index (*r* =  − 0.197) and serum albumin (*r* =  − 0.188), but higher MIS (*r* = 0.176). Higher levels of TG *n*-3 polyunsaturated fatty acids (PUFAs) were associated with lower MIS (*r* =  − 0.168) and interleukin-6 concentrations (*r* =  − 0.115). Higher levels of TG *n*-6 PUFAs were associated with lower BMI (*r* =  − 0.149) but greater serum albumin (*r* = 0.112). In conclusion, TG monounsaturated fatty acids were associated with poor nutritional status, while TG *n*-3 PUFAs were associated with good nutritional status. On the other hand, TG saturated fatty acids and TG *n*-6 PUFAs had both favorable and unfavorable associations with nutritional parameters.

## Introduction

The stage of chronic kidney disease (CKD) marked by an irreversible glomerular filtration rate decline to 20–30 ml/min/1.73 m^2^ in a patient, essentially favors development of metabolic acidosis, retention of uremic toxins and generation of pro-inflammatory cytokines^1,2^. The net uremic burden is a chronic inflammatory milieu, which is further enhanced in end stage kidney disease patients on dialysis^1^. Indeed, inflammatory markers such as C-reactive protein (CRP) and interleukin-6 (IL-6) are strongly associated with cardiovascular and all-cause mortality in patients on maintenance hemodialysis (HD)^3,4^. Separately, inflammation is also implicated in protein energy wasting (PEW), a state of decreased body stores of protein and fat^5^. Causal mechanisms for PEW implicate appetite suppression, increased energy expenditure, insulin resistance, and skeletal muscle degradation^6^. The scope of PEW in global dialysis populations indicates a prevalence range between 28 and 54%^7^. Of concern, the loss of muscle and fat stores in PEW-affected HD patients has been associated with increased mortality^8–10^.

The involvement of inflammation in the pathology of both PEW and cardiovascular disease is unexplained and raises a query if *there is any interrelationship between these outcomes?* One plausible pathway would involve circulating fatty acids (FAs) status that are linked to cardiovascular events and mortality in both non-CKD^11^ and dialysis patients^12^, via the modulation of inflammatory response pathways^13^. In individuals without kidney disease, associations between blood FA levels and inflammatory markers are evident^14–19^. In these individuals, circulating *n*-3 and *n*-6 polyunsaturated fatty acids (PUFAs) were observed to be inversely associated with CRP and IL-6 levels^14–16^, whilst saturated fatty acids (SFAs) and monounsaturated fatty acids (MUFAs) were directly associated with CRP and IL-6 levels^17–19^.

In the context of dialysis patients, evidence is scarce on circulating FA profiles relating to indices of inflammation, nutritional status, and body composition. To date three studies on HD patients have examined relationships of circulating PUFAs with inflammatory markers (CRP and IL-6) and nutritional status [body mass index (BMI), serum albumin, and subjective global assessment score]^20–22^. However, these studies excluded circulating SFAs and MUFAs in relation to markers of inflammation, nutritional status or body composition. In the context that insulin resistance is also involved in the pathogenesis of PEW, associations between circulating FAs and insulin resistance remains unexplored.

In a primary analysis of data from the Malaysian Palm Tocotrienols in Chronic Hemodialysis (PaTCH), we reported that plasma triglyceride (TG) FAs were reflective of dietary PUFA intakes^23^. However, Farzaneh-Far et al.^24^ suggested that examining circulating FA status is relevant to interpreting health outcomes rather than the dietary FA intake, as substantial individual variability occurs with dietary FA absorption and metabolism. Given the gap in knowledge relating to circulating FA status, inflammation and nutritional status for patients on HD, we aimed to use the comprehensive database of the PaTCH HD population to investigate and explore our research question. Our hypothesis was that the complete FA profile carried by plasma TG-FAs would be differentially associated with nutritional status, body composition, inflammatory markers, and insulin resistance in HD patients.

## Methods and materials

### Patients and study design

This study is a cross-sectional analysis of data collected during the screening of eligible patients for the PaTCH study as previously described^23^. In brief, patients were recruited from 11 HD centers from October 2015 to October 2016. Eligible patients were those aged > 18 years old, dialyzed thrice-weekly for more than 3 months, and able to provide fasting blood samples. Patients with poor adherence to HD regime, mental and physical disability, or concomitant serious illness such as infection with human immunodeficiency virus or acquired immune deficiency syndrome or malignancies were excluded. This study has received ethical approval from the Research Ethics Committee of National University of Malaysia (NN-078-2015) and the Medical Research Ethics Committee of the Ministry of Health, Malaysia (NMRR-15-865-25260). All patients provided written informed consent and all research procedures were conducted in accordance with relevant guidelines and regulations.

### Sociodemographic and lifestyle assessment

Patients’ sociodemographic data, medical history, recent drug prescription, and routine laboratory parameters were collected from medical records. The International Physical Activity Questionnaire (short version)^25^ was included for assessment of physical activity level. The result is reported as a continuous variable, namely metabolic equivalent (MET) minutes a week, which represents the amount of energy expended on physical activity.

### Nutritional status assessment

#### Anthropometry

Post-dialysis dry weight and height, measured using a calibrated digital weighing scale and a portable stadiometer (SECA 213, SECA Corporation, Deutschland, Germany) respectively, were used to derive patients’ BMI. Waist circumference (WC) and mid-arm circumference (MAC) was measured using a non-stretch measuring tape (Lufkin W606PM, Apex Tool Group, Maryland, USA) while triceps skinfold (TSF) thickness was measured using a Harpenden skinfold caliper (John Bull, British Indicator, UK) on the non-fistula arm. Mid-arm muscle circumference (MAMC) and mid-arm muscle area (MAMA) were derived from MAC and TSF using the equation of Heymsfield et al.^26^:

$$\begin{gathered} {\text{MAMC}}\,\left( {{\text{cm}}} \right) = {\text{MAC}}\,\left( {{\text{cm}}} \right){-}{\text{TSF}}\,\left( {{\text{cm}}} \right) \times\uppi \hfill \\ {\text{MAMA}}\,\left( {{\text{cm}}^{{2}} } \right) = {\text{MAMC}}\,\left( {{\text{cm}}} \right)^{{2}} /{4}\uppi {-}{1}0.0\,\left( {{\text{for}}\,{\text{men}}} \right)\,{\text{or}}\,{6}.{5}\,\left( {{\text{for}}\,{\text{women}}} \right) \hfill \\ \end{gathered}$$

All anthropometric measurements were performed in accordance to the protocol of the International Society for the Advancement of Kinanthropometry^27^ by a single trained researcher to avoid inter-observer bias.

#### Biochemistry

Approximately 10 ml fasting blood was collected via the dialysis access of a patient into EDTA and Lithium Heparin tubes (Becton Dickinson Vacutainer, NJ, USA) during a mid-week pre-dialysis session. Blood samples were immediately centrifuged at 3000 rpm for 10 min and plasma aliquots were snap frozen in liquid nitrogen and stored at – 80 °C freezer until further analyses.

Plasma concentration of high sensitivity C-reactive protein (hsCRP) was measured using automated particle enhanced immunoturbidimetric assay (Cobas, Roche Diagnostics, Indiana, USA) with a measuring range between 0.15 and 20.0 mg/L. Plasma IL-6 was analyzed manually using commercial sandwich enzyme-linked immunosorbent assay kits (ab46042 High Sensitivity IL-6 Human Elisa, Abcam, UK), with a detection range between 1.56 and 50.0 pg/mL. The kits were read on a microplate reader (iMark Microplate Absorbance Reader, Bio-Rad Laboratories, California, USA), using 450 nm as the primary wavelength. Samples for hsCRP and IL-6 analysis were diluted for reruns whenever the upper limit of measurement was exceeded. The cut-off values indicating an activated inflammatory response for hsCRP and IL-6 were 10 mg/L^3^ and 5.9 pg/mL^4^, respectively.

Serum albumin was analyzed by the bromocresol green method, plasma glucose by an enzymatic method (glucose oxidase) while fasting insulin levels were measured using electrochemiluminescence. These analyses were carried out by an independent laboratory using an automated clinical chemistry analyzer (Roche/Hitachi 912 System, Roche Diagnostics, Tokyo, Japan). The Homeostasis Model Assessment of Insulin Resistance (HOMA-IR) was derived^28^ using the following formula:

$${\text{HOMA - IR}} = \left[ {{\text{serum}}\,{\text{insulin}}\,\left( {\upmu {\text{U}}/{\text{mL}}} \right) \times {\text{plasma}}\,{\text{glucose}}\,\left( {{\text{mmol}}/{\text{L}}} \right)} \right]/{22}.{5}$$

Plasma TG-FAs were determined using gas chromatography as previously described^23,29^. Briefly, lipids from plasma were extracted using a chloroform–methanol mixture (2:1). Plasma lipids were then separated into lipid components by thin layer chromatography (TLC) with a mixed solvent phase of hexane, diethyl ether, and acetic acid (80:20:2). The TG band isolated from the TLC plates (Silica gel 60, Merck, Darmstadt, Germany) were converted into fatty acid methyl esters and reconstituted with hexane before injection into the gas chromatographer (Shimadzu GC-2010, Shimadzu Corporation, Japan) installed with a 100 m capillary column (SP-2560, Supelco, USA). Individual FAs were identified by comparing their peak retention times with known standards (Supelco-37 Component FAME Mix, Supelco, Bellefonte, USA), and concentrations were expressed as a percentage of total peak area^23^.

#### Body composition and physical strength

Patients’ lean tissue index (LTI) and fat tissue index (FTI) were assessed by bio-impedance spectroscopy using the Body Composition Monitor (BCM; Fresenius Medical Care, Germany) before the dialysis session as per manufacturer’s instructions to avoid the issue of post-dialysis fluid redistribution. The BCM device provides output values of overhydration, lean tissue mass and fat tissue mass based on the three-compartment model of body composition. Both LTI and FTI are lean tissue and fat tissue masses normalized to height squared, respectively^30^. A hand dynamometer (Jamar Plus+, Sammons Preston, Illinois, USA) was used to assess patients’ handgrip strength^31^ before the dialysis session. Patients were asked to squeeze the dynamometer with maximum pressure, using their non-fistula arm with elbow flexed at 90°. Three measurements were taken at 10-s intervals and the median value was used for analysis.

#### Dietary assessment

Patients’ dietary energy and protein intakes were assessed using the 3-day dietary recall method inclusive of a dialysis, a non-dialysis and a weekend day^32^. Trained dietitians conducted the assessment through face-to-face interviews and household measurement tools were utilized to optimize portion size recalls^33^.

#### Assessment of protein energy wasting

The diagnostic criteria proposed by the International Society of Renal Nutrition and Metabolism (ISRNM) Expert Group^5^ were used to assess PEW. A positive assessment was indicated by the presence of 3 of the following 4 criteria: serum albumin < 38 g/L, BMI < 23 kg/m^2^, reduction > 10% in MAMC in relation to the 50th percentile of a reference population^34^, or dietary energy intake < 25 kcal/kg ideal body weight.

The malnutrition–inflammation score (MIS) was also used for diagnosis of PEW^35^. The MIS consists of 10 components, each scored from 0 (normal) to 3 (very severe) with a final combined score ranging from 0 to 30. A higher MIS score reflects a greater severity of malnutrition and inflammation and MIS score ≥ 5 are indicative of PEW^7^.

### Statistical analyses

The Shapiro–Wilk test was used to assess normality of data. Normally distributed continuous variables are presented as mean ± SD while non-normal distributed continuous variables are presented as median with interquartile range (IQR). Categorical variables are presented as frequency (percentages). We analyzed four major FA classes, namely SFA, MUFA, *n*-3 PUFA, and *n*-6 PUFA as well as 11 individual FAs, including C12:0 (lauric acid), C14:0 (myristic acid), C16:0 (palmitic acid), C18:0 (stearic acid), C16:1*n*-7 (palmitoleic acid), C18:1 (oleic acid), C18:2*n*6 (linoleic acid, LA), C20:4*n*6 (arachidonic acid), C18:3*n*3 (α-linolenic acid, ALA), C20:5*n*3 (eicosapentaenoic acid, EPA), and C22:6*n*3 (docosahexaenoic acid, DHA). All TG-FAs were log-transformed and reported as geometrical means with 95% confidence intervals. Independent *t*-test and Mann–Whitney test were used to compare normally distributed and non-normally distributed variables, respectively, for patients with and without PEW. Chi-square test was used to determine the association between categorical variables and PEW while Pearson’s correlation was used to determine the association between continuous variables. Multivariate linear regression analyses were used to determine the associations between TG-FAs (independent variables) and dependent variables inclusive of nutritional parameters (BMI, WC, TSF, MAMA, LTI, FTI, handgrip strength, and MIS) and biochemical markers (hsCRP, IL-6, albumin, insulin, and HOMA-IR). Separate regression analyses were performed for both FA classes as well as individual FAs to avoid multiple collinearity. For example, the four main FA groups, namely SFA, MUFA, *n*-6 PUFA, and *n*-3 PUFA were included in one model whilst all individual FAs were analyzed in another model. Variance inflation factor was used to check for multiple collinearity. The analyses were adjusted with potential confounding factors such as age, gender, dialysis vintage, Kt/V, Charlson comorbidity index, total calorie intake, prescription of statin, dialysis access, and MET-score (for physical activity level). Dependent variables and covariates with skewed distribution were log-transformed before analyses. All analyses were computed using the IBM SPSS version 26.0 (IBM SPSS Statistics Inc., Chicago, IL, USA). Statistical significance was set at *p* < 0.05 for all evaluated parameters.

## Results

**Figure 1:**
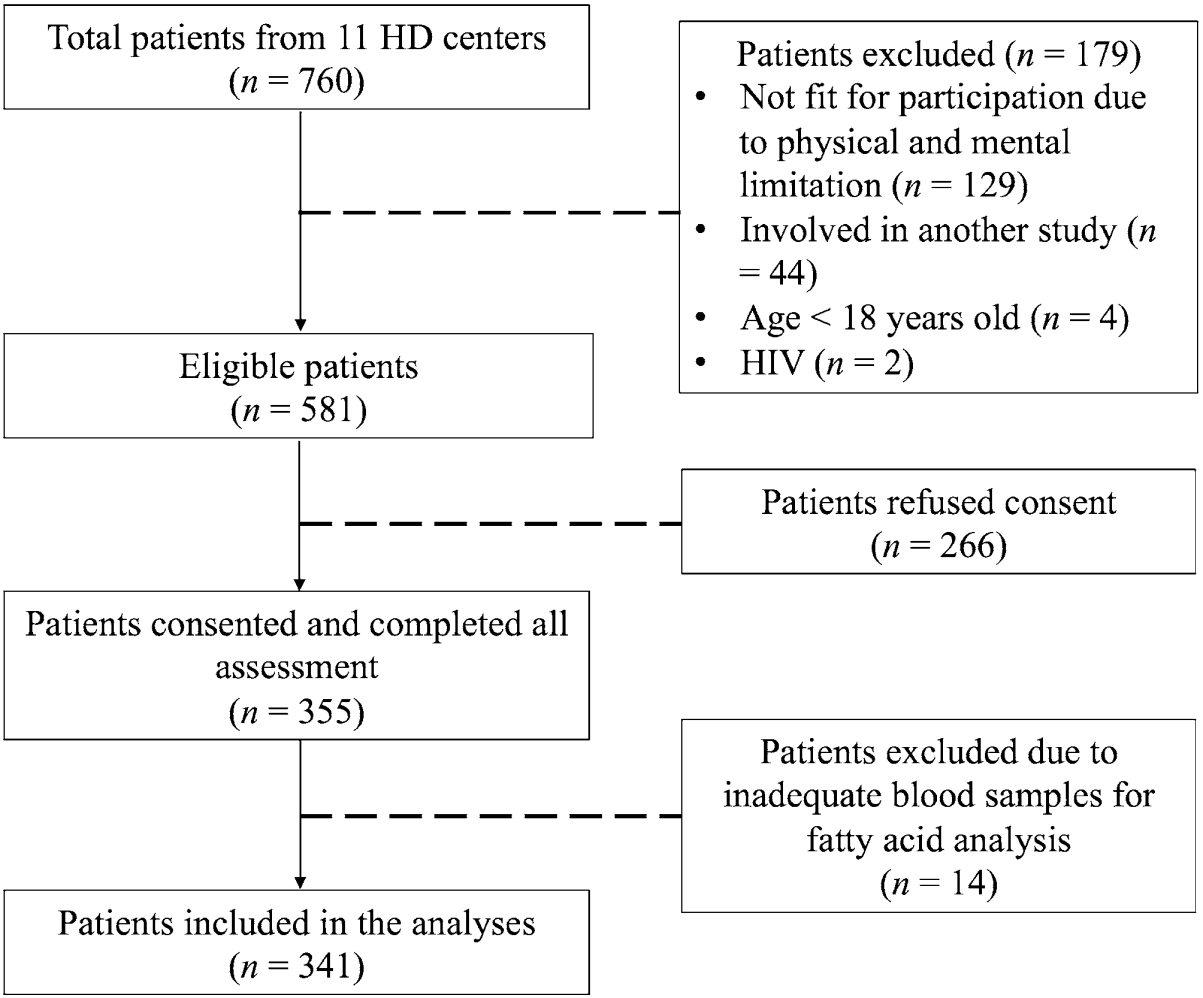
Flow chart of patient enrollment and analysis in this study.

The final analyses included 341 HD patients (Fig. 1) and their baseline characteristics are shown in Table 1. The mean age was 55.2 ± 14.0 years, with an ethnic distribution of 53.7% Chinese, 29.0% Malay and 16.4% Indian; 54.3% were male and 41.9% were diabetic. The median dialysis vintage was 64 months (IQR: 80 months). For inflammatory status, median values for hsCRP and IL-6 were 7.4 mg/L (IQR: 6.8 mg/L) and 5.9 pg/mL (IQR: 3.8 pg/mL) respectively, with 19.6% of patients having hsCRP ≥ 10 mg/L and 27.3% with IL-6 ≥ 5.9 pg/mL.

**Table 1:** Patient baseline characteristics and comparison between patients with and without protein-energy wasting.

Variables	All (*n* = 341)	PEW (*n* = 202)	Non PEW (*n* = 139)
Age, year	55.2 ± 14.0	56.3 ± 13.3	53.7 ± 14.8
**Sex, n (%)**			
Male	185 (54.3)	103 (51.0)	82 (59.0)
Female	156 (45.7)	99 (49.0)	57 (41.0)
**Ethnicity, n (%)**			
Malay	99 (29.0)	54 (26.7)	45 (32.4)
Chinese	183 (53.7)	108 (53.5)	75 (54.0)
Indian	56 (16.4)	39 (19.3)	17 (12.2)
Others	3 (0.9)	2 (1.4)	1 (0.5)
Dialysis vintage, month	64 (80)*	79 (85)	37 (68)^†^
**Dialysis access, n (%)**			
Fistula	294 (86.2)	170 (84.2)	124 (89.2)
Catheter	47 (13.8)	32 (15.8)	15 (10.8)
Charlson Comorbidities Index	4 (3)*	4 (3)	4 (3)
Smokers, *n* (%)	29 (8.5)	16 (7.9)	13 (9.4)
Prescription of statin, *n* (%)	205 (60.1)	126 (62.4)	79 (56.8)
Dry weight, kg	61.3 ± 14.5	58.5 ± 15.6	65.5 ± 11.7^†^
Height, cm	158.1 ± 8.8	157.6 ± 8.9	158.8 ± 8.6
BMI, kg/m^2^	24.5 ± 4.9	23.4 ± 5.2	25.9 ± 0.3^†^
≥ 23 kg/m^2^	207 (60.7)	98 (48.5)	36 (25.9)^†^
< 23 kg/m^2^	134 (39.3)	104 (51.5)	103 (74.1)
Triceps skinfold, mm^a^	15.9 (10.1)*	14.9 (9.2)	18.4 (10.6)^†^
Mid arm muscle area, cm^2,a^	36.3 (15.0)*	34.2 (16.6)	38.9 (12.9)^†^
Lean tissue index, kg/m^2,b^	12.7 (3.6)*	12.2 (3.5)	13.3 (3.5)^†^
Fat tissue index, kg/m^2,b^	11.2 (6.8)*	10.5 (6.6)	12.0 (6.4)^†^
MIS	5 (5)*	3 (2)	7 (17)^†^
Serum pre-dialysis urea, mmol/L	19.5 ± 5.5	18.9 ± 5.8	20.5 ± 5.1^†^
Serum pre-dialysis creatinine, μmol/L	814 (262)*	769 (244)	863 (245)^†^
Serum phosphorus, mmol/L	1.79 ± 0.52	1.72 ± 0.51	1.88 ± 0.52^†^
Serum albumin, g/L	39.1 ± 3.9	38.1 ± 4.1	40.7 ± 3.0^†^
Hemoglobin, g/L	10.8 ± 1.7	10.7 ± 1.7	10.8 ± 1.6
Plasma glucose, mmol/L^c^	5.4 (2.0)*	5.3 (1.5)	5.4 (2.4)
Serum insulin, μU/mL^c^	9.5 (9.3)*	7.8 (8.5)	11.1 (13.1)^†^
HOMA-IR^c^	2.3 (3.0)*	2.0 (2.3)	2.8 (4.8)^†^
Kt/V	1.7 ± 0.4	1.7 ± 0.4	1.6 ± 0.4^†^
hsCRP, mg/L	7.4 (6.8)*	4.3 (7.4)	3.1 (4.6)^†^
< 10 mg/L	274 (80.4)	157 (77.7)	117 (84.2)
≥ 10 mg/L	67 (19.6)	45 (22.3)	22 (15.8)
IL-6, pg/mL^d^	5.9 (3.8)*	4.2 (5.4)	3.4 (2.9)^†^
< 5.9 pg/mL	240 (72.7)	130 (67.0)	110 (80.9)^†^
≥ 5.9 pg/mL	90 (27.3)	64 (33.0)	26 (19.1)
Energy intake, kcal/kg/day	25.2 ± 5.5	25.1 ± 5.2	25.3 ± 5.9
Protein intake, g/kg/day	0.9 ± 0.3	0.9 ± 0.3	0.9 ± 0.3
Fish oil supplement	6 (1.8)	3 (1.5)	3 (2.2)
MET-minutes	198 (586)*	99 (413)	297 (792)^†^

Protein energy wasting is defined as malnutrition inflammation score ≥ 5.

*Values are expressed as median (interquartile range).

^†^Analyses indicate *p *value < 0.05 for comparison between patients with and without protein-energy wasting based on independent *t*-test, Mann–Whitney test, or Chi-square test.

^a^*n* = 340, ^b^*n* = 336, ^c^*n* = 304, ^d^*n* = 331.

hsCRP, high-sensitivity C-reactive protein; HOMA-IR, homeostatic model assessment of insulin resistance; IL-6, interleukin-6; MET, metabolic equivalent; MIS, malnutrition inflammation score; PEW, protein-energy wasting.

The plasma TG-FA profiles of patients are presented in Table 2. All identified total TG-FAs were greater than 95% of total fatty acid composition. The major TG-FA subclass was MUFA (45.1%), followed by SFA (35.5%) and PUFA (16.7%). The proportion of TG-*n*-6 PUFA (15.6%) was more than 20-fold greater than TG-*n*-3 PUFA (0.7%). In relation to individual FAs, the most abundant TG-FA was oleic (41.4%), followed by palmitic (29.1%) and LA (14.3%).

**Table 2:** Fatty acid composition of individual and classes of plasma triglyceride.

Fatty acid	Geometric means (95% CI)
**SFA**	35.45 (35.09–35.80)
12:0	0.27 (0.22–0.33)
14:0	1.24 (1.18–1.29)
16:0	29.08 (28.79–29.34)
18:0	3.50 (3.42–3.59)
**MUFA**	45.06 (44.70–45.42)
16:1*n*7	2.85 (2.94–2.96)
18:1*n*9	41.43 (41.10–41.76)
**PUFA**	16.69 (16.38–17.00)
*n*-6 PUFA	15.58 (15.30–15.85)
18:2*n*6	14.27 (14.01–14.54)
20:4*n*6	0.70 (0.61–0.81)
*n*-3 PUFA	0.66 (0.58–0.74)
18:3*n*3	0.19 (0.15–0.24)
20:5*n*3	0.022 (0.019–0.026)
22:6*n*3	0.15 (0.12–0.18)

Fatty acid values are percentage of total fatty acids and presented as geometric means (95% confidence interval).

CI, confidence interval; MUFA, monounsaturated fatty acid; PUFA, polyunsaturated fatty acid; SFA, saturated fatty acid.

Fatty acid nomenclature: 12:0 (lauric acid), 14:0 (myristic acid), 16:0 (palmitic acid), 16:1*n*-7 (palmitoleic acid), 18:0 (stearic acid), 18:1 (oleic acid), 18:2*n*6 (linoleic acid), 18:3*n*3 (α-linolenic acid), 20:4*n*6 (arachidonic acid), 20:5*n*3 (eicosapentaenoic acid), 22:6*n*3 (docosahexaenoic acid).

**Figure 2:**
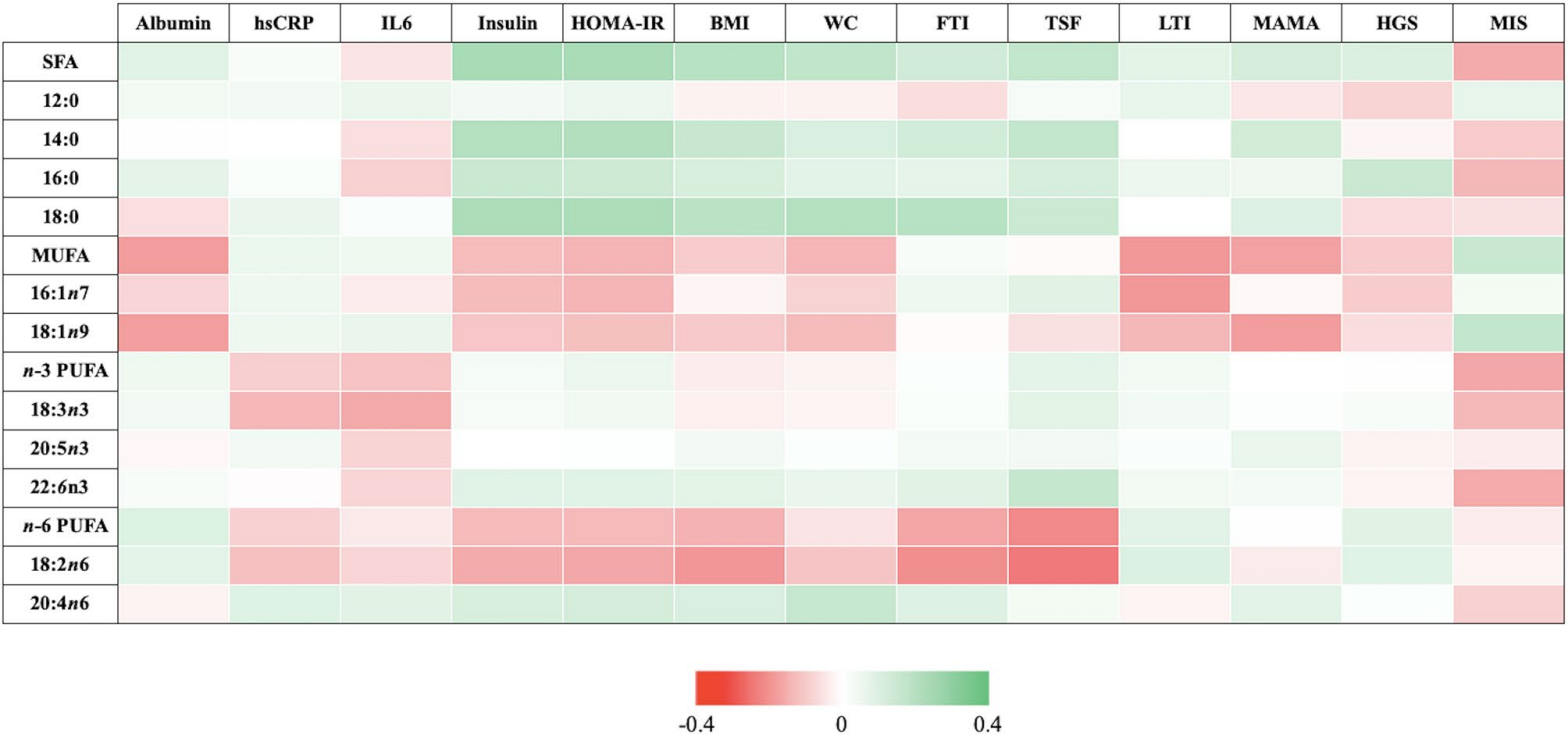
Correlation matrices between triglyceride fatty acids and biochemical markers, body composition, physical strength, and nutritional status. Correlations are represented by different colour cells: the red scale (*r* = 0 to − 0.4) indicates negative associations while the green scale (*r* = 0 to 0.4) indicates positive associations.

Figure 2 and Supplementary Table S1 indicate the correlation matrices between TG-FAs and biochemical markers, body composition, physical strength, and nutritional status. Overall, TG-SFA and individual TG-SFAs such as lauric, palmitic and stearic acids were positively associated with glycemic markers (serum insulin and HOMA-IR), indicators of adiposity (BMI, waist circumference, FTI, and TSF), MAMA, and handgrip strength. In contrast, TG-*n*-6 PUFAs and TG-LA showed negative associations with these glycemic markers and indicators of adiposity whilst the opposite association was observed for TG-arachidonic acid. In contrast, TG-MUFAs and TG-oleic acid were negatively associated with serum albumin, glycemic markers (serum insulin and HOMA-IR), and muscle mass measures (LTI and MAMA), but positively associated with MIS. Of note, TG-*n*-3 PUFAs and TG-ALA were negatively associated with inflammatory markers (hsCRP and IL-6) and MIS.

The associations of plasma TG-FAs with biochemical markers and nutritional parameter, namely MIS, body composition, and physical strength are presented in Tables 3 and 4, respectively. Key observations were:

- TG-SFA was positively associated with BMI (*β* = 9.225, *p* = 0.024), FTI (*β* = 1.262, *p* = 0.006), TSF (*β* = 0.890, *p* = 0.019), serum insulin (*β* = 2.428, *p* < 0.001), and HOMA-IR (*β* = 2.798, *p* < 0.001) but negatively associated with MIS (*β* = − 1.113, *p* = 0.028). Amongst individual SFAs, TG-lauric acid was positively associated with MIS (*β* = 0.038, *p* = 0.021), TG-myristic acid was positively associated with serum insulin (*β* = 0.396, *p* = 0.001), and HOMA-IR (*β* = 0.440, *p* = 0.001), TG-palmitic acid was positively associated with HOMA-IR (*β* = 1.140 *p* = 0.047), while TG-stearic acid as directly associated with waist circumference (*β* = 6.527, *p* = 0.049) and FTI (*β* = 0.345, *p* = 0.019).
- TG-MUFA was negatively associated with LTI (*β* = − 0.584, *p* = 0.004). For individual MUFAs, TG-palmitoleic acid was negatively associated with LTI (*β* = − 0.081, *p* = 0.020), serum insulin (*β* = − 0.374, *p* = 0.006), and HOMA-IR (*β* = − 0.454, *p* = 0.003), while TG-oleic acid was negatively associated with MAMA (*β* = − 0.944, *p* = 0.009) and serum albumin (*β* = − 9.751, *p* = 0.006) but positively associated with MIS (*β* = 1.101, *p* = 0.030).
- TG-*n*-3 PUFA was negatively associated with IL-6 (*β* = − 0.096, *p* = 0.031) and MIS (*β* = − 0.068, *p* = 0.022). Amongst individual *n*-3 PUFAs, TG-ALA was negatively associated with hsCRP (*β* = − 0.074, *p* = 0.019), and IL-6 (*β* = − 0.062, *p* = 0.007).
- TG-*n*-6 PUFA was positively associated with handgrip strength (*β* = 0.410 *p* = 0.025). Amongst individual *n*-6 PUFAs, TG-LA was negatively associated with BMI (*β* = − 4.715, *p* = 0.019) and TSF (*β* = − 0.459, *p* = 0.014) while TG-arachidonic acid was positively associated with waist circumference (*β* = 1.177, *p* = 0.017) and IL-6 (*β* = 0.078, *p* = 0.033).

**Table 3:** Summary of multivariate linear regression analyses for associations between plasma TG fatty acids with biochemical variables.

TG fatty acid	Albumin	log hsCRP	log IL-6	log Insulin	log HOMA-IR
	*β (p-value)*	*β (p-value)*	*β (p-value)*	*β (p-value)*	*β (p-value)*
**Total FA**^**a**^					
SFA	4.198 (0.25)	0.252 (0.81)	− 0.957 (0.22)	**2.428 (< 0.001)**	**2.798 (< 0.001)**
MUFA	− 5.235 (0.21)	0.410 (0.74)	− 0.447 (0.62)	− 0.052 (0.95)	− 0.225 (0.80)
*n*-3 PUFA	0.054 (0.80)	− 0.050 (0.41)	** − 0.096 (0.031)**	0.053 (0.18)	0.083 (0.06)
*n*-6 PUFA	2.62 (0.25)	− 0.162 (0.78)	− 0.222 (0.60)	− 0.015 (0.97)	− 0.092 (0.82)
*r*^*2*^	0.030	0.022	0.081	0.165	0.242
**Individual FA**^**b**^					
12:0	− 0.020 (0.87)	0.045 (0.20)	0.031 (0.22)	− 0.008 (0.72)	− 0.001 (0.98)
14:0	0.311 (0.63)	− 0.141 (0.45)	− 0.159 (0.24)	**0.396 (0.001)**	**0.440 (0.001)**
16:0	1.498 (0.60)	0.252 (0.76)	− 1.096 (0.07)	0.889 (0.08)	**1.140 (0.047)**
18:0	− 1.742 (0.13)	0.388 (0.24)	− 0.101 (0.68)	0.273 (0.19)	0.211 (0.37)
16:1*n*7	− 1.082 (0.14)	0.373 (0.08)	− 0.057 (0.72)	** − 0.374 (0.006)**	** − 0.454 (0.003)**
18:1*n9*	** − 9.751 (0.006)**	0.808 (0.43)	− 0.028 (0.97)	− 0.464 (0.47)	− 0.652 (0.37)
18:2*n*6	− 0.263 (0.88)	− 0.062 (0.90)	− 0.548 (0.15)	− 0.300 (0.35)	− 0.414 (0.26)
20:4*n*6	− 0.033 (0.85)	0.079 (0.12)	**0.078 (0.033)**	0.033 (0.39)	0.036 (0.32)
18:3*n*3	0.050 (0.65)	** − 0.074 (0.019)**	** − 0.062 (0.007)**	0.017 (0.38)	0.031 (0.16)
20:5*n*3	− 0.179 (0.35)	0.058 (0.29)	− 0.039 (0.33)	− 0.018 (0.61)	− 0.020 (0.62)
22:6*n*3	0.077 (0.56)	− 0.010 (0.79)	− 0.028 (0.32)	0.022 (0.35)	0.026 (0.34)
*r*^*2*^	0.044	0.105	0.021	0.197	0.266

Multivariate linear regression analyses adjusted for age, gender, dialysis vintage, Kt/V, Charlson Comorbidity Index, prescription of statin, dialysis access, and metabolic equivalent-score.

^a^The analysis model was concurrently inclusive of all fatty acid groups and the highest variance inflation factor was 3.178.

^b^The analysis model was concurrently inclusive of all individual fatty acids and the highest variance inflation factor was 3.473.

Values in bold indicate *p*-value < 0.05.

FA, fatty acid, HOMA-IR, homeostatic model assessment of insulin resistance; hsCRP, high-sensitivity C-reactive protein; IL-6, interleukin-6; SFA, saturated fatty acid; TG, triglyceride.

Fatty acid nomenclature: 12:0 (lauric acid), 14:0 (myristic acid), 16:0 (palmitic acid), 16:1*n*-7 (palmitoleic acid), 18:0 (stearic acid), 18:1 (oleic acid), 18:2*n*6 (linoleic acid), 18:3*n*3 (α-linolenic acid), 20:4*n*6 (arachidonic acid), 20:5*n*3 (eicosapentaenoic acid), 22:6*n*3 (docosahexaenoic acid).

**Table 4:** Summary of multivariate linear regression analyses for associations between plasma TG fatty acids with body composition, physical strength, and nutritional status.

TG fatty acid	BMI	WC	log LTI	log FTI	log TSF	log MAMA	log HGS	log MIS
	*β (p-value)*	*β (p-value)*	*β (p-value)*	*β (p-value)*	*β (p-value)*	*β (p-value)*	*β (p-value)*	*β (p-value)*
**Total FA**^a^								
SFA	**9.225 (0.024)**	20.26 (0.06)	− 0.159 (0.35)	**1.262 (0.006)**	**0.890 (0.019)**	0.180 (0.61)	0.606 (0.07)	** − 1.113 (0.028)**
MUFA	− 2.502 (0.60)	− 6.941 (0.59)	** − 0.584 (0.004)**	0.801 (0.14)	0.090 (0.84)	− 0.670 (0.11)	0.305 (0.44)	0.157 (0.79)
*n*-3 PUFA	− 0.237 (0.31)	− 0.650 (0.26)	0.001 (0.95)	0.010 (0.70)	0.041 (0.06)	− 0.029 (0.16)	0.012 (0.53)	** − 0.068 (0.022)**
*n*-6 PUFA	− 1.936 (0.38)	− 0.381 (0.95)	− 0.080 (0.38)	− 0.083 (0.74)	− 0.389 (0.06)	0.019 (0.92)	**0.410 (0.025)**	− 0.214 (0.43)
Adjusted *r*^*2*^	0.212	0.287	0.301	0.208	0.274	0.141	0.336	0.137
**Individual FA**^b^								
12:0	− 0.154 (0.25)	− 0.295 (0.38)	0.009 (0.10)	− 0.021 (0.16)	− 0.002 (0.90)	− 0.018 (0.12)	− 0.015 (0.18)	**0.038 (0.021)**
14:0	1.232 (0.10)	1.377 (0.45)	0.006 (0.84)	0.064 (0.44)	0.066 (0.34)	0.089 (0.17)	0.013 (0.84)	− 0.119 (0.19)
16:0	0.996 (0.75)	− 3.283 (0.68)	− 0.057 (0.67)	0.426 (0.23)	0.460 (0.12)	− 0.186 (0.50)	0.444 (0.10)	− 0.345 (0.38)
18:0	1.937 (0.14)	**6.527 (0.049)**	− 0.026 (0.64)	**0.345 (0.019)**	0.203 (0.10)	0.010 (0.93)	0.021 (0.85)	− 0.112 (0.49)
16:1*n*7	− 1.135 (0.17)	− 1.063 (0.60)	** − 0.081 (0.020)**	0.009 (0.92)	− 0.037 (0.64)	0.022 (0.76)	0.043 (0.54)	0.070 (0.50)
18:1*n9*	− 5.417 (0.19)	− 18.76 (0.08)	− 0.302 (0.08)	0.185 (0.69)	− 0.143 (0.71)	** − 0.944 (0.009)**	− 0.162 (0.64)	**1.101 (0.030)**
18:2*n*6	** − 4.715 (0.019)**	− 9.422 (0.06)	− 0.028 (0.74)	− 0.380 (0.09)	** − 0.459 (0.014)**	− 0.120 (0.49)	0.289 (0.09)	0.047 (0.85)
20:4*n*6	0.273 (0.17)	**1.177 (0.017)**	− 0.013 (0.11)	0.028 (0.22)	− 0.016 (0.40)	0.024 (0.17)	− 0.009 (0.60)	− 0.008 (0.76)
18:3*n*3	− 0.053 (0.67)	− 0.135 (0.66)	0.005 (0.33)	0.008 (0.54)	0.019 (0.11)	− 0.003 (0.79)	0.006 (0.59)	− 0.030 (0.06)
20:5*n*3	− 0.113 (0.59)	− 0.630 (0.22)	0.000 (0.97)	− 0.011 (0.65)	− 0.005 (0.79)	0.006 (0.76)	0.001 (0.96)	0.036 (0.17)
22:6*n*3	− 0.064 (0.67)	− 0.311 (0.39)	0.005 (0.45)	− 0.004 (0.82)	0.023 (0.10)	− 0.019 (0.15)	0.010 (0.41)	− 0.033 (0.07)
Adjusted *r*^*2*^	0.219	0.305	0.305	0.217	0.277	0.141	0.329	0.148

Multivariate linear regression analyses adjusted for age, gender, dialysis vintage, Kt/V, Charlson Comorbidity Index, energy intake, dialysis access, and metabolic equivalent-score.

^a^The analysis model was concurrently inclusive of all fatty acid groups and the highest variance inflation factor was 3.096.

^b^The analysis model was concurrently inclusive of all individual fatty acids and the highest variance inflation factor was 3.400.

Values in bold indicate *p*-value < 0.05.

BMI, body mass index; FA, fatty acid; FTI, fat tissue index; HGS, handgrip strength; LTI, lean tissue index; MAMA, mid-arm muscle area; MIS, malnutrition inflammation score; MUFA, monounsaturated fatty acid; PUFA, polyunsaturated fatty acid; SFA, saturated fatty acid; TSF, tricep skinfold; WC, waist circumference.

Fatty acid nomenclature: 12:0 (lauric acid), 14:0 (myristic acid), 16:0 (palmitic acid), 16:1*n*-7 (palmitoleic acid), 18:0 (stearic acid), 18:1 (oleic acid), 18:2*n*6 (linoleic acid), 18:3*n*3 (α-linolenic acid), 20:4*n*6 (arachidonic acid), 20:5*n*3 (eicosapentaenoic acid), 22:6*n*3 (docosahexaenoic acid).

The comparison of TG-FA profile between patients with and without PEW is shown in Table 5. The key observations were:

- Patients diagnosed with PEW based on the ISRNM criteria had significantly lower TG-SFA (*p* = 0.006), TG-myristic (*p* = 0.047), and TG-palmitic acid (*p* = 0.001).
- Patients diagnosed with PEW based on MIS had significantly lower TG-SFA (*p* = 0.005), TG-myristic acid (*p* = 0.026), TG-*n*-3 PUFA (*p* = 0.003), TG-ALA (*p* = 0.004), and TG-DHA (*p* = 0.014) but higher TG-MUFA (*p* = 0.006) and TG-oleic acid (*p* = 0.004).

**Table 5:** Plasma TG fatty acid status comparisons between PEW and non-PEW patients.

Fatty acids	ISRNM PEW criteria			MIS		
	PEW (*n* = 73)	Non-PEW (*n* = 268)	*P* value^a^	PEW^b^ (*n* = 139)	Non-PEW (*n* = 202)	*P* value^a^
**SFA**	34.7 (3.4)	35.6 (3.9)	**0.006**	35.0 (3.7)	35.9 (3.9)	**0.005**
12:0	0.3 (1.0)	0.3 (0.9)	0.475	0.3 (0.8)	0.4 (1.1)	0.554
14:0	1.2 (0.5)	1.3 (0.7)	**0.047**	1.2 (0.7)	1.3 (0.7)	**0.026**
16:0	28.4 (3.1)	29.5 (3.0)	**0.001**	29.0 (3.1)	29.6 (3.1)	0.096
18:0	3.5 (1.0)	3.5 (1.0)	0.564	3.5 (1.0)	3.6 (0.9)	0.299
**MUFA**	45.4 (4.5)	45.1 (3.8)	0.473	45.6 (3.7)	44.6 (4.1)	**0.006**
16:1*n*7	2.9 (1.7)	2.9 (1.7)	0.742	3.0 (1.7)	2.8 (1.6)	0.612
18:1*n*9	41.8 (3.9)	41.7 (3.4)	0.387	42.0 (3.3)	41.2 (4.1)	**0.004**
**PUFA**	16.4 (3.7)	16.9 (3.7)	0.934	16.7 (3.7)	16.9 (3.8)	0.354
*n*-6 PUFA	15.3 (3.7)	15.6 (3.2)	0.955	15.4 (3.0)	15.6 (3.4)	0.696
18:2*n*6	14.3 (3.2)	14.3 (3.2)	0.386	14.2 (3.0)	14.4 (3.5)	0.787
20:4*n*6	0.9 (0.6)	0.9 (0.5)	0.093	0.9 (0.5)	0.9 (0.5)	0.791
*n*-3 PUFA	0.7 (1.0)	0.9 (1.0)	0.606	0.7 (1.0)	1.0 (0.9)	**0.003**
18:3*n*3	0.3 (0.5)	0.4 (0.4)	0.873	0.3 (0.4)	0.4 (0.4)	**0.004**
20:5*n*3	0.01 (0.06)	0.01 (0.05)	0.988	0.01 (0.05)	0.01 (0.06)	0.731
22:6*n*3	0.2 (0.6)	0.3 (0.7)	0.078	0.2 (0.6)	0.4 (0.7)	**0.014**

Data is expressed as median (interquartile range).

^a^Mann–Whitney test.

^b^PEW is defined as MIS ≥ 5.

Values in bold indicate *p*-value < 0.05.

ISRNM, International Society of Renal Nutrition and Metabolism; MIS, malnutrition inflammation score; MUFA, monounsaturated fatty acid; PEW, protein energy wasting; PUFA, polyunsaturated fatty acid; SFA, saturated fatty acid.

FA nomenclature: 12:0 (lauric acid), 14:0 (myristic acid), 16:0 (palmitic acid), 16:1*n*-7 (palmitoleic acid), 18:0 (stearic acid), 18:1 (oleic acid), 18:2*n*6 (linoleic acid), 18:3*n*3 (α-linolenic acid), 20:4*n*6 (arachidonic acid), 20:5*n*3 (eicosapentaenoic acid), 22:6*n*3 (docosahexaenoic acid).

## Discussion

In this cross-sectional study, we examined associations between TG-FAs and biomarkers of inflammation, insulin resistance and PEW in maintenance HD patients. Within the established FA profile of circulating lipids, TG-MUFA appeared to be associated with unfavorable outcomes in relation to body composition and PEW status, whilst TG-*n*-3 PUFAs were favorably associated with lower inflammatory markers and better nutritional assessment parameters of the patients. Notably we found that TG-*n*-6 PUFAs were associated with lower BMI and body fat of the patients but greater physical strength as demonstrated by handgrip strength, whilst TG-SFAs were associated with greater body fat reserves and insulin resistance.

In relation to inflammation, TG-*n*-3 PUFAs, specifically TG-ALA, was found to be inversely associated with hsCRP and IL-6. Circulatory ALA levels reflect dietary origins since humans are unable to synthesize ALA^36^. Our findings on the association between TG-ALA and inflammatory markers are consistent with previous studies in non-CKD populations^37,38^ and in agreement with data on ALA supplementation and CRP levels in HD patients^39^. A review of in vitro studies hypothesized that the mechanism of anti-inflammatory properties of ALA involves (i) inhibition of the nuclear factor-κB pathway via activation of peroxisome proliferator-activated receptor-γ (ii) inactivation of the NLRP3 inflammasome and (iii) attenuation of the pro-inflammatory phenotype of M1-like macrophages^40^. In addition, plasma ALA competes with LA for the same enzymes in the PUFA biosynthesis pathway, which fosters lower synthesis of pro-inflammatory eicosanoids^41^. A prospective cohort study observed that a higher dietary *n*-6/*n*-3 PUFA ratio intake in HD patients was associated with increased inflammation over time and mortality^42^, suggesting the relative amount of dietary *n*-6 and *n*-3 PUFAs is critical in modulation of inflammatory response of HD patients. Of note, we observed the association of TG-arachidonic acid only with IL-6 in our HD patients, and it is pertinent to appreciate that this FA is synthesized from LA via desaturation and elongation process, and serves as a precursor for the production of pro-inflammatory eicosanoids^43^.

In contrast to our findings on TG-ALA and inflammation, phospholipid-LA was instead shown to be significantly associated with reduced inflammatory markers in Swedish HD patients^20^, while, no associations between CRP and any plasma long chain PUFAs were noted in a cohort of Japanese HD patients^21^. Such differences with our study may be attributed to study methodology and patient population. First, these studies assessed plasma phospholipid^20^ and total plasma FA^21^, whereas TG-FAs were evaluated in our study. Second, both Swedish and Japanese HD patients plasma FA profiles had higher *n*-3 and *n*-6 PUFA levels compared to our samples, arising from greater fish consumption and use of different fats and oils^12^. We did not observe any significant association between inflammatory markers and TG-EPA and TG-DHA in our study population, who had suboptimal plasma levels of these FAs^23^.

As per nutritional status, TG-SFAs were associated with higher BMI, which in the scenario of ‘reverse epidemiology’ is associated with improving survival outcome in CKD populations^44^. A similar association between plasma SFA and BMI was reported in a non-CKD Lebanese population^45^. Inspection of our data revealed that both TG-SFAs were separately and directly associated with body fat mass as opposed to lean tissue mass. Although low fat mass has been associated with increased risk of mortality in HD patients^10^, the distribution of fat mass is an important determinant of risk because waist circumference, (a surrogate measure of central obesity), was associated with higher all-cause and cardiovascular mortality in dialysis patients^46^. In the present study, total TG-SFA and TG-stearic acid were associated with increased waist circumference. In contrast, higher TG-LA levels were associated with reduced BMI and triceps skinfold thickness, although no association with lean tissue mass was apparent. Interestingly, TG-*n*-6 PUFA was associated with increased handgrip strength, indicating the inverse association with triceps skinfold did not affect physical strength. A prospective study would be required to elucidate the relationship between plasma FAs, body composition and clinical outcomes in HD patients.

In terms of insulinemic status, we observed that overall TG-SFA was associated with increased serum insulin and HOMA-IR but this association was only limited to TG-myristic and TG-palmitic acids but not TG-lauric and TG-stearic acids. Similar to our study, high serum SFA was reported to be associated with insulin resistance in pre-dialysis CKD patients but individual SFA breakdown data were not shown^47^. A meta-analysis of prospective cohort studies on non-CKD populations showed that only circulating myristic acid, not other SFAs, was associated with incident type 2 diabetes mellitus^48^. Although plasma SFAs are hypothesized to be synthesized from the de novo lipogenesis pathway, plasma myristic acid appears to be a minor product^48^. Therefore, circulating myristic acid is likely of dietary origin and our previous study also observed a non-significant trend (*p* = 0.056) for the association between dietary and TG-myristic acid^23^. The association between TG-myristic acid and insulin resistance may be also linked to obesity, as obesity measured by BMI is correlated with insulin resistance^49^.

Based on the MIS cutoff ≥ 5^7^, PEW was present in 59% of the HD patients in this study. A high MIS composite score is strongly associated with dialysis mortality compared to serum albumin alone (per 1 g/dL decrease)^35^. Since both BMI and albumin are the components of MIS with the opposite relationship to it, the positive association between TG-MUFAs and MIS observed in our study was quite predictable. Similarly, TG-*n*-3 PUFA and TG-ALA were also associated with lower MIS, which may be attributed to the effect of *n*-3 PUFAs on modulation of inflammatory response as discussed earlier. A randomized controlled trial demonstrated that HD patients receiving *n*-3 PUFA supplementation for 12 weeks significantly improved their MIS rating compared to a placebo group^50^.

Generally, TG-MUFAs were associated with unfavorable nutritional status and body composition represented by lower serum albumin, MAMA and LTI values, as well as higher MIS. Son et al.^51^ reported that HD patients with significant vascular calcification score exhibited enhanced erythrocyte MUFA and oleic acid content. Plasma nervonic acid, another MUFA, has been separately associated with increased mortality in a small cohort of dialysis patients^52^. Therefore, enhanced circulating MUFA levels may be associated with unfavorable clinical outcomes in HD patients. However, this does not imply that dietary MUFA intake is detrimental as plasma MUFA is also synthesized endogenously, and we showed previously for this same population that plasma MUFA levels bore a poor correlation with dietary MUFA^23^. In fact, HD patients tend to have greater plasma MUFA levels compared to healthy controls, which is likely attributed to the uremic impact on fatty acid metabolism resulting in reduced circulating levels of *n*-3 and *n*-6 PUFA^12^.

The current study has several strengths. First, different subclasses of FAs in plasma TG were measured directly using gas chromatography and the total percentage of identified plasma TG-FAs was greater than 95%. Second, we included two inflammatory markers and assessed a comprehensive range of confounders possibly affecting the inflammation^53^ and nutritional status, including age, smoking, medical history, physical activity level, medication use, and dialysis access, which were factored into the analyses. In addition, the inflammatory biomarkers, nutritional parameters, and body composition assessment that were included in the assessment, have robust prediction outcomes in HD populations. Third, one trained researcher performed all anthropometric measurements thereby minimizing measurement bias. Our study also has some limitations. First, the cross-sectional association cannot establish the causality of observations. Although a reverse causation is possible, the explanation is biologically less plausible as some interventional studies have demonstrated the potential effects of FA in modulating inflammatory status and clinical outcomes^24^. Second, the findings based on plasma TG-FA status may not be applicable to erythrocyte or other lipid fractions. Third, this study focused on HD patients in Malaysia and results may not be applicable to other HD populations as dietary consumption patterns invariably differ. Lastly, performance of bio-impedance analysis before the dialysis treatment could potentially introduce noise related to fluid retention when measuring LTI.

In conclusion, plasma TG *n*-3 PUFAs were associated with lower levels of inflammatory markers and better nutritional status in patients undergoing maintenance HD. Contrarily, plasma TG-SFA, specifically myristic acid, was associated with increased BMI, waist circumference, body fat mass, and insulin resistance whilst *n*-6 PUFAs were associated with lower triceps skinfold but greater handgrip strength. Plasma TG-MUFAs were associated with poor nutritional status and reduced lean tissue mass. Interventional studies are warranted to confirm the potential effects of dietary fat quality manipulation on nutritional status, inflammatory profiles, and clinical endpoints in HD patients.

## Supplementary Information

Supplementary Table.

## Data Availability

The datasets generated and/or analyzed during this study are available on reasonable request from the corresponding author, T.K.
