# Supplementary material for: Circulating fatty acid profiles are associated with protein energy wasting in maintenance hemodialysis patients: a cross-sectional study
Source: Sci Rep. 2021 Jan 14;11:1416. doi: 10.1038/s41598-020-80812-1 (PMC7809126; doi:10.1038/s41598-020-80812-1)
Supplement: Supplementary file 1 — Supplementary Table. [file 41598_2020_80812_MOESM1_ESM.docx]

**Circulating Fatty Acid Profiles are Associated with Protein Energy Wasting in Maintenance Hemodialysis Patients: A Cross-sectional Study**

Ban-Hock Khor^1^, Sharmela Sahathevan^2^, Ayesha Sualeheen^2^, Mohammad Syafiq Md Ali^3^, Sreelakshmi Sankara Narayanan^4^, Karuthan Chinna^5^, Abdul Halim Abdul Gafor^1^, Bak-Leong Goh^6^, Ghazali Ahmad^7^, Zaki Morad^8^, Zulfitri Azuan Mat Daud^3^, Pramod Khosla^9^, Kalyana Sundram^10^, Tilakavati Karupaiah^4,*^, PaTCH Investigators

^1^Department of Medicine, Faculty of Medicine, Universiti Kebangsaan Malaysia Medical Center, Kuala Lumpur 56000, Malaysia; khorbanhock@gmail.com (B.-H.K.), halimgafor@gmail.com

^2^Dietetics Program, Faculty of Health Sciences, Universiti Kebangsaan Malaysia, Kuala Lumpur 50300, Malaysia; sham_0901@yahoo.com (S.S.), aishaltaf@ymail.com (A.S.),

^3^Department of Nutrition and Dietetics, Faculty of Medicine and Health Sciences, Universiti Putra Malaysia, Selangor 43400, Malaysia; mohammadsyafiqali14@gmail.com (M.S.M.A.), zulfitri@upm.edu.my (Z.A.M.D.)

^4^School of BioSciences, Faculty of Health and Medical Sciences, Taylor's University, Subang Jaya, Selangor 47500, Malaysia; sreelakshmiprem@hotmail.com (S.S.N.), tilly_karu@hotmail.co.uk (T.K.)

^5^School of Medicine, Faculty of Health and Medical Sciences, Taylor’s University, Subang Jaya, Selangor 47500, Malaysia; karuthan@gmail.com

^6^Clinical Research Center, Hospital Serdang, Selangor 43000, Malaysia; bak.leong@gmail.com

^7^Department of Nephrology, Hospital Kuala Lumpur, Kuala Lumpur 53000, Malaysia; ghazaliahmad2018@gmail.com

^8^National Kidney Foundation of Malaysia, Petaling Jaya, Selangor 46100, Malaysia; zakimorad@gmail.com

^9^Department of Nutrition and Food Sciences, Wayne State University, Detroit, MI 48202, USA; aa0987@wayne.edu

^10^Malaysia Palm Oil Council, Kelana Jaya, 47301 Malaysia; kalyana@mpoc.org.my

*Correspondence: Tilakavati Karupaiah, School of Biosciences, Faculty of Health and Medical Sciences, Taylor’s University, Subang Jaya 47500, Malaysia. E-mail: [tilly_karu@yahoo.co.uk](mailto:tilly_karu@yahoo.co.uk)

Table S1 Correlation matrices between triglyceride fatty acids and biochemical markers, body composition, physical strength, and nutritional status

| TG FA | Albumin | hsCRP | IL6 | Insulin | HOMA-IR | BMI | WC | FTI | TSF | LTI | MAMA | HGS | MIS |
| --- | --- | --- | --- | --- | --- | --- | --- | --- | --- | --- | --- | --- | --- |
| SFA | 0.099 | 0.023 | -0.052 | 0.280^**^ | 0.276^**^ | 0.230^**^ | 0.203^**^ | 0.150^**^ | 0.197^**^ | 0.091 | 0.135^*^ | 0.118^*^ | -0.160^**^ |
| 12:0 | 0.042 | 0.045 | 0.070 | 0.045 | 0.064 | -0.025 | -0.026 | -0.063 | 0.032 | 0.074 | -0.046 | -0.081 | 0.078 |
| 14:0 | 0.009 | 0 | -0.062 | 0.246^**^ | 0.247^**^ | 0.179^**^ | 0.124^*^ | 0.151^**^ | 0.194^**^ | 0.001 | 0.153^**^ | -0.018 | -0.100 |
| 16:0 | 0.086 | 0.022 | -0.089 | 0.172^**^ | 0.162^**^ | 0.128^*^ | 0.094 | 0.083 | 0.133^*^ | 0.064 | 0.054 | 0.171^**^ | -0.136^*^ |
| 18:0 | -0.059 | 0.068 | 0.017 | 0.268^**^ | 0.265^**^ | 0.218^**^ | 0.244^**^ | 0.230^**^ | 0.167^**^ | 0.002 | 0.108^*^ | -0.067 | -0.058 |
| MUFA | -0.188^**^ | 0.066 | 0.053 | -0.126^*^ | -0.144^*^ | -0.095 | -0.140^*^ | 0.027 | -0.008 | -0.197^**^ | -0.178^**^ | -0.098 | 0.176^**^ |
| 16:1*n*7 | -0.078 | 0.059 | -0.034 | -0.129^*^ | -0.142^*^ | -0.019 | -0.083 | 0.060 | 0.095 | -0.198^**^ | -0.011 | -0.095 | 0.038 |
| 18:1*n*9 | -0.184^**^ | 0.056 | 0.071 | -0.108 | -0.119^*^ | -0.103 | -0.129^*^ | -0.007 | -0.057 | -0.136^*^ | -0.188^**^ | -0.064 | 0.193^**^ |
| *n*-3 PUFA | 0.053 | -0.094 | -0.115^*^ | 0.034 | 0.062 | -0.035 | -0.022 | 0.013 | 0.089 | 0.043 | 0.003 | 0.009 | -0.168^**^ |
| 18:3*n*3 | 0.045 | -0.139^**^ | -0.163^**^ | 0.031 | 0.050 | -0.031 | -0.021 | 0.022 | 0.092 | 0.040 | 0.011 | 0.024 | -0.134^*^ |
| 20:5*n*3 | -0.015 | 0.045 | -0.082 | 0.006 | 0.007 | 0.040 | 0.014 | 0.036 | 0.045 | 0.018 | 0.070 | -0.022 | -0.035 |
| 22:6*n*3 | 0.026 | -0.004 | -0.077 | 0.097 | 0.098 | 0.091 | 0.068 | 0.095 | 0.188^**^ | 0.042 | 0.037 | -0.020 | -0.161^**^ |
| *n*-6 PUFA | 0.112^*^ | -0.089 | -0.040 | -0.130^*^ | -0.133^*^ | -0.149^**^ | -0.051 | -0.171^**^ | -0.227^**^ | 0.093 | 0.008 | 0.096 | -0.034 |
| 18:2*n*6 | 0.087 | -0.120^*^ | -0.078 | -0.157^**^ | -0.167^**^ | -0.199^**^ | -0.110^*^ | -0.214^**^ | -0.254^**^ | 0.113^*^ | -0.037 | 0.100 | -0.020 |
| 20:4*n*6 | -0.022 | 0.107^*^ | 0.096 | 0.125^*^ | 0.135^*^ | 0.124^*^ | 0.185^**^ | 0.108^*^ | 0.038 | -0.020 | 0.093 | 0.015 | -0.089 |

Abbreviations: BMI, body mass index; FA, fatty acid; FTI, fat tissue index; HGS, handgrip strength; HOMA-IR, homeostatic model assessment of insulin resistance; hsCRP, high sentivity C-reactive protein; IL-6, interleukin-6; LTI, lean tissue index; MAMA, mid-arm muscle area; MIS, malnutrition inflammation score; MUFA, monounsaturated fatty acid; PUFA, polyunsaturated fatty acid; SFA, saturated fatty acid; TG, triglyceride; TSF, triceps skinfold; WC, waist circumference.

Fatty acid nomenclature: 12:0 (lauric acid), 14:0 (myristic acid), 16:0 (palmitic acid), 16:1*n*-7 (palmitoleic acid), 18:0 (stearic acid), 18:1 (oleic acid), 18:2*n*6 (linoleic acid), 18:3*n*3 (α-linolenic acid), 20:4*n*6 (arachidonic acid), 20:5*n*3 (eicosapentaenoic acid), 22:6*n*3 (docosahexaenoic acid).

Note: ***p*<0.01, **p*<0.05. All TG-FAs, hsCRP, IL-6, insulin, HOMA-IR, FTI, TSF, LTI, MAMA, HGS, and MIS were log-transformed prior to statistical analyses.
